# Serum from Calorie-Restricted Rats Activates Vascular Cell eNOS through Enhanced Insulin Signaling Mediated by Adiponectin

**DOI:** 10.1371/journal.pone.0031155

**Published:** 2012-02-02

**Authors:** Fernanda M. Cerqueira, Laura I. Brandizzi, Fernanda M. Cunha, Francisco R. M. Laurindo, Alicia J. Kowaltowski

**Affiliations:** 1 Instituto de Química, Departamento de Bioquímica, Universidade de São Paulo, São Paulo, São Paulo, Brazil; 2 Instituto do Coração, Faculdade de Medicina, Universidade de São Paulo, São Paulo, São Paulo, Brazil; 3 Escola de Artes, Ciências e Humanidades, Universidade de São Paulo, São Paulo, São Paulo, Brazil; Pennington Biomedical Research Center, United States of America

## Abstract

eNOS activation resulting in mitochondrial biogenesis is believed to play a central role in life span extension promoted by calorie restriction (CR). We investigated the mechanism of this activation by treating vascular cells with serum from CR rats and found increased Akt and eNOS phosphorylation, in addition to enhanced nitrite release. Inhibiting Akt phosphorylation or immunoprecipitating adiponectin (found in high quantities in CR serum) completely prevented the increment in nitrite release and eNOS activation. Overall, we demonstrate that adiponectin in the serum from CR animals increases NO^•^ signaling by activating the insulin pathway. These results suggest this hormone may be a determinant regulator of the beneficial effects of CR.

## Introduction

Calorie restriction (CR) extends lifespans of model organisms ranging from yeast to mammals [1]–[4], and many groups have focused on understanding how this dietary intervention acts mechanistically. In 2005, Nisoli and collaborators [5] elegantly demonstrated that dietary restriction induced the activation of endothelial nitric oxide synthase (eNOS) and lead to enhanced mitochondrial biogenesis and increased oxygen consumption. Indeed, the effects of the diet were largely absent in eNOS deficient animals [5]. Further studies have found links between mitochondrial activity and CR. Fungal CR models present increments in respiratory activity [6]–[8], and CR in yeast can be promoted by NO^•^-stimulated mitochondrial biogenesis [9]. Furthermore, CR prevents the decline in respiratory activity seen in aging rats [10], [11] and increasing respiratory activity through the use of mitochondrial uncouplers enhances mouse lifespan [12]. Interestingly, both CR and uncouplers enhance mitochondrial biogenesis in insulin-sensitive tissues, in a manner involving protein kinase B (Akt) phosphorylation [13].

Insulin is involved in the control of eNOS phosphorylation and activity [14]–[18]. It activates Akt [17], [19], [20], which promotes eNOS activation [21], increasing the production of nitric oxide (NO^•^) and leading to mitochondrial biogenesis [22]–[25] through the expression of the peroxisome proliferator-activated receptor-γ coactivator 1α (PGC-1α), a master regulator of mitochondrial mass (reviewed in [26], [27]).

The mechanism which leads to NO^•^ signaling and mitochondrial biogenesis in response to CR was not well explored to date. Mammals submitted to CR present lower insulin levels [13], [28], [29], but improved tissue insulin sensitivity [13], [30], in part due to long-term decreases in blood glucose [31]. We investigate here if changes in serological profiles in CR animals are sufficient to acutely promote NO^•^ signaling in cultured vascular cells, and uncover the signaling pathways involved.

## Results

### CR decreases serum glucose and insulin; increases adiponectin levels

After 26 weeks of CR, the average body weight of rats was lower than control AL rats, an effect accompanied by lower visceral fat deposits, serum glucose, insulin, and increased adiponectin levels (Table 1), alterations similar to those observed in most literature CR studies [28].

**Table 1:** Effects of CR and AL diets.

	CR	AL	P value
Body weight (g)	481.5±82.9	675.7±93.1	<0.0001
Visceral fat (g)	23.9±9.8	31.2±7.9	0.0009
Serum glucose (mg⋅dL^−1^)	85.8±3.7	115.1±6.6	0.0008
Serum insulin (ng⋅mL^−1^)	0.58±0.29	1.98±0.85	<0.0001
Serum adiponectin (relative to AL)	3.0±0.7	1	<0.0001

Measurements were conducted as described in Materials and Methods.

### CR serum increases NO^•^ production

**Figure 1:**
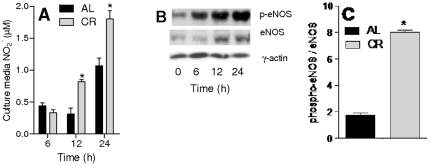
CR serum increases NO_2_^−^ release and promotes eNOS and nNOS phosphorylation. (**A**) Culture media NO_2_^−^ was measured over time after incubation in AL or CR sera, as indicated. (**B**) eNOS phosphorylation and expression over time after switching from AL to CR serum. Representative blots are shown. (**C**). Quantification of eNOS phosphorylation after 24 h in AL or CR sera. *p<0.05 versus AL.

VSMC cells incubated in media in which standard serum was substituted for serum collected from CR rats presented a time-dependent increase in NO_2_^−^, indicative of higher levels of NO^•^ production compared to cells maintained in media containing serum from animals fed AL (Fig. 1A). This result shows that acute treatment with serum from CR animals is sufficient to increase VSMC NO^•^ production, and suggests CR serum contains regulatory signals leading to this effect.

We sought to determine the source of this augmented NO^•^ production by measuring the activities of eNOS in cells which had been cultured in AL media and were then switched to media containing serum from CR animals. Under these conditions, the quantity of total eNOS increased significantly after 24 h (by 203±8%, p<0.05). Furthermore, active, phosphorylated, eNOS increased (Fig. 1B shows a representative blot of the time-dependent effect of incubation in CR serum, while Fig. 1C quantifies relative phosphorylated band intensity after 24 h in AL or CR sera). Overall, these results indicate that eNOS expression and activation is promoted by serological changes induced by CR.

### CR serum increases insulin signaling

**Figure 2:**
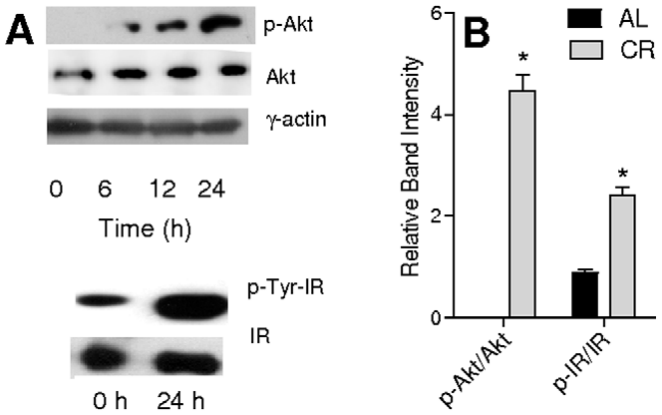
CR serum activates the insulin pathway. (**A**) Upper blots: Akt^Ser473^ phosphorylation over time after switching from AL to CR serum. Lower blots: Tyr phosphorylation in insulin receptors (IR) immunoprecipitated from VSMC cultured for 24 h in AL or CR media. Representative blots are shown. (**B**) Quantification of pAkt/Akt and p-IR/IR after 24 h in AL or CR sera. *p<0.05 versus AL.

We have previously shown that Akt and eNOS are activated in insulin-sensitive tissues of CR animals [13]. We sought to measure the activity of this pathway in VSMC cells cultured in the presence of CR serum (Fig. 2) and found that the active, phosphorylated, form of Akt increased in a time-dependent manner in CR media, from undetectable levels in AL serum (Fig. 2A, upper panels). Indeed, after 24 h in CR serum, a highly significant change in p-Akt levels was detected relative to AL serum (Fig. 2B).

Among other pathways controlling Akt, this protein is sensitive to insulin signaling. Although insulin levels in CR serum are decreased relative to AL (Table 1), we measured the activation of insulin receptors (IR) from VSMC grown 24 h in AL and CR media. The receptors were immunoprecipitated and probed with anti-phospho-Tyr antibodies. CR serum significantly enhanced the total amount of IR by 194±8%, p<0.05, and lead to a strong increment in receptor phosphorylation (Figs. 2A and 2B), indicating that it contains components other than insulin capable of acutely activating the insulin pathway.

### CR serum-induced NO^•^ release is dependent on Akt

**Figure 3:**
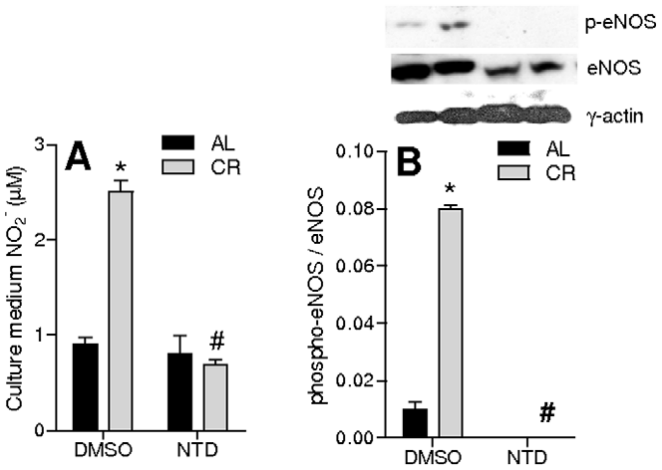
CR-induced NO_2_^−^ release is dependent on Akt activity. (**A**) NO_2_^−^ levels in the culture medium from VSMC incubated 24 h with AL or CR serum and 0.001% DMSO (solvent control) or 1 µM NTD. (**B**) eNOS^Ser1177^ phosphorylation in homogenates from VSMC incubated 24 h with AL or CR serum and 0.001% DMSO or 1 µM NTD. *p<0.05 versus AL; ^#^p<0.05 versus DMSO. Representative blots are shown above quantifications.

In order to investigate if enhanced NO^•^ release from VSMC cells was dependent on the activation of the insulin pathway, we inhibited Akt activity with 1 µM naphthyridinone 17 (NTD). This concentration of NTD completely prevented the accumulation of NO_2_^−^ promoted by CR serum, but did not affect the release in cells grown in AL serum (Fig. 3A). Furthermore, NTD completely eliminated the detection of phospho-eNOS and decreased total eNOS band intensity (Fig. 3B). This is consistent with the finding that Akt activity is important for eNOS phosphorylation [21].

### Adiponectin mediates the activation of the insulin pathway and NO^•^ release induced by CR serum

**Figure 4:**
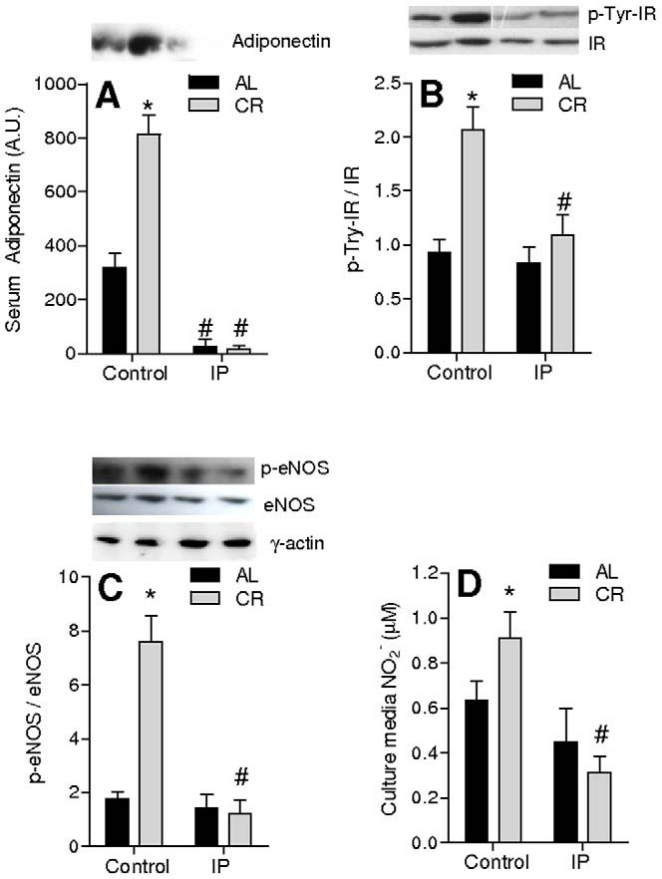
Adiponectin in CR serum promotes insulin receptor and eNOS phosphorylation, resulting in NO^•^ release. (**A**) Adiponectin levels in AL and CR sera before and after immunoprecipitation (IP). (**B**) Tyr phosphorylation in insulin receptors immunoprecipitated from homogenates of VSMC cultured for 24 h in the presence of CR or AL serum, with (IP) or without (Control) prior adiponectin immunoprecipitation. (**C**) eNOS^Ser1177^ phosphorylation in homogenates from VSMC cultured for 24 h in the presence of CR or AL serum, with (IP) or without (Control) prior adiponectin immunoprecipitation. (**D**) NO_2_^−^ levels in the media from VSMC cultured for 24 h in the presence of CR or AL serum, with (IP) or without (Control) prior adiponectin immunoprecipitation. *p<0.05 versus AL; ^#^p<0.05 versus control. Representative blots, shown above quantifications, were cut to remove other bands, without any further image manipulation.

The activation of the insulin pathway in cells acutely treated with CR serum is surprising since insulin levels are lower (Table 1). However, adiponectin levels are increased in CR, and this hormone is an activator of the insulin pathway [32], [33]. To address the role of adiponectin in the CR serum effect on NO^•^ release, we removed it through immunoprecipitation. The procedure was highly effective (Fig. 4A). Using immunoprecipitated sera, we noted that the phosphorylation of insulin receptors promoted by CR serum was eliminated (Fig. 4B), while no effect was seen in AL serum. Immunoprecipitation of adiponectin also totally reversed the effect of CR serum on eNOS phosphorylation (Fig. 4C) and on NO_2_^−^ release (Fig. 4D). Overall, these results indicate that enhanced NO^•^ release promoted by CR serum in vascular cells is a consequence of high adiponectin levels.

## Discussion

Mitochondrial mass and function decrease during aging [34]–[36] in a manner prevented by CR, which promotes enhanced NO^•^ signaling associated with mitochondrial biogenesis [5], [6], [11], [13]. Thus, NO^•^ signaling seems to be central toward the beneficial effects of CR in aging, although the mechanisms through which CR affects this pathway have not been directly approached to date. We addressed this point by treating VSMC, prone to respond to physiological stimuli that affect NO^•^ release [37], [38], with serum collected from CR animals. This protocol has the advantage of separating long-term dietary effects from acute effects on vascular cells, specifically addressing the question if hormonal changes in CR are sufficient to activate NO^•^ signaling.

We observed a time-dependent increment in NO_2_^−^ released into the culture medium, indicative of enhanced NO^•^ production, as well as increments in eNOS quantity and phosphorylation (Fig. 1), a result in line with previous data showing that CR induced the expression of eNOS through Akt [5], [13], [17], [19], [20]. Indeed, Akt phosphorylation was strongly enhanced by CR serum (Fig. 2) and NTD (a selective Akt inhibitor when used at low micromolar doses [39]) inhibited eNOS phosphorylation (Fig. 3).

The insulin receptor, an upstream regulator of Akt activity and eNOS activation [40], was also activated by CR serum (Fig. 2). Insulin signaling is well known to activate NO^•^ signaling, and Akt physically interacts with eNOS in response to insulin [41]. However, insulin is found at decreased levels in CR serum while adiponectin, an activator of peripheral insulin signaling [32], [33], is increased (Table 1, [42], [43]). Furthermore, adiponectin was previously reported to activate eNOS through Akt [44], [45].

Accordingly, we sought to determine if adiponectin in CR serum could activate NO^•^ signaling. We immunoprecipitated adiponectin from both AL and CR sera (Fig. 4A), and found that, while this did not alter the release of NO_2_^−^ promoted by AL serum, it completely abrogated the increased release specific to CR serum (Fig. 4D). In addition, increased activation of the insulin pathway and eNOS were absent upon removal of adiponectin (Figs. 4B and C). Together, these results demonstrate that adiponectin is the key regulator of enhanced NO^•^ signaling in vascular cells stimulated with CR serum.

It should be noted that VSMCs present different signaling receptors and pathways than endothelial cells, which could thus present different responses to CR sera. However, previous results demonstrate that adiponectin stimulates NO^•^ release from endothelial cells [46], supporting the idea that this cytokine is probably a key signaling molecule in CR-induced NO^•^ signaling. Interestingly, it seems that eNOS-derived NO^•^ can also have a determinant role in regulating the production of adiponectin by adipocytes [47].

Overall, our results point to adiponectin as a key serological factor involved in acute cellular responses altered by CR, and suggest that this hormone may be a central regulator of mitochondrial biogenesis and other processes involving NO^•^ signaling.

## Materials and Methods

### Animals and serum collection

All experiments were conducted in agreement with National Institutes of Health guidelines for humane treatment of animals and were approved (unnumbered) the local Animal Care and Use Committee (*Comissão de Ética em Cuidados e Uso Animal*). Male, 8-week-old Sprague-Dawley rats were separated into 2 groups: AL, fed *ad libitum* with an AIN-93-M diet prepared by Rhoster (Campinas, SP, Brazil) and CR, fed at levels 60% of AL ingested amounts a diet supplemented with micronutrients to reach the vitamin and mineral levels consumed by AL animals [48]. Food was offered daily at 6 pm and feedings were adjusted weekly by weight, based on AL food consumption. The intervention resulted in known alterations associated with CR including lower body weight and improved insulin sensitivity [49]. The animals were lodged 3 per cage and given water *ad libitum*. At 34 weeks (26 weeks on the diet), rats were sacrificed after 12 hours fasting and the serum was obtained as described in [50], allowed to clot for 20–30 min at 25°C and centrifuged for 20 min at 300 *g*. The supernatant was collected and stored (−20°C). Sera were thawed and heat-inactivated at 56°C for 30 min prior to use.

### Serum analysis

Insulin, glucose, triglycerides, HDL, total cholesterol and adiponectin levels from AL or CR sera were evaluated (Table 1). Peripheral blood was collected from the tail of 40-week-old animals fasted for 12 hours and used for glucose analysis (Accu-Check® Performa Glucose Analyzer, São Paulo, SP, Brazil). For insulin and adiponectin determinations, blood samples were centrifuged at 1000 *g* for 15 min and the supernatant was stored at −20°C. Insulin was measured using a Linco Research ELISA kit (St. Charles, MO, USA). Adiponectin was detected by Western Blots.

### Cell cultures

Rat vascular smooth muscle cells (VSMC) were purchased from ATCC (CRL-2797™) and cultured in 25 mM glucose DMEM supplemented with 18 mM sodium bicarbonate, 4 mM glutamine, 0.3 mM geneticin, 100 µg/mL streptomycin, 100 U/mL penicillin and 10% v/v fetal bovine serum, at 37°C and 5% CO_2_. Cells were passaged every 3 days. After the 8^th^ passage, cells were cultured in medium where fetal bovine serum was substituted for AL rat serum. After 2 further passages, cells from a 70% confluent flask were washed and cultured in DMEM with CR or AL rat sera. Where used, naphthyridinone 17 (NTD) was pre-incubated with the cultures for 24 hours, while the control group was incubated with the same quantity of the solvent DMSO.

After 6, 12 or 24 hours, cell culture media were removed and stored at −80°C for NO_2_^−^ measurements. Cells were washed, detached and counted in a Newbauer chamber. The cells were then centrifuged (300 *g*, 5 min, 4°C) and homogenized in 50 mM Tris-HCl buffer, pH 7.4, supplemented with 1% glycerol, 10% protease inhibitor cocktail (Sigma), 1% octyl phenol ethoxylate, 10 mM sodium orthovanadate, 10 mM sodium fluoride and 10 mM sodium pyrophosphate. After 30 min over ice, cell lysates were centrifuged (13,000 *g*, 20 min, 4°C) and the resulting supernatants were collected.

### NO_2_^−^ levels

NO_2_^−^, a marker of NO^•^ levels [51], was measured using an NO^•^ analyzer (Model 208A; Sievers Instruments Inc., Boulder, CO, USA) according to manufacturer protocols through the detection of chemiluminescence in the presence of potassium iodide and acetic acid [52], [53]. Basal NO_2_^−^ levels from the media were subtracted.

### Western Blots

Total proteins from cell lysates or serum were diluted in Laemmli sample buffer (100 mM Tris.HCl, 2% w/v SDS, 10% v/v glycerol, 0.1% bromophenol blue) containing 100 mM dithiothreitol, with the exception of eNOS and phospho-eNOS Western Blots, which were performed without dithiothreitol. After heating at 90°C for 5 min, proteins were separated by SDS-PAGE and transferred onto nitrocellulose membranes. Membranes were blocked with 5% BSA and detection was carried out using specific primary antibodies against: Adiponectin (Abcam, 1∶2,000); eNOS (Sigma, 1∶3,000); phospho-eNOS^Ser1177^ (Cell Signaling, C9C3 clone, 1∶1,000); Akt (Calbiochem, 1∶1,000), phospho-Akt^Ser473^ (Cell Signaling, 1∶3,000); and γ-actin (Sigma, 1∶2,000). Chemiluminescent detection was performed using a secondary peroxidase-linked anti-rabbit (Calbiochem, 1∶10,000) or anti-sheep IgG (Calbiochem, 1∶13,000) and a detection system from Pierce KLP (Rockford, IL, USA). The specificity of anti-NOS antibodies [54] was determined by molecular mass comparisons. Signals were quantified by densitometry using ImageQuant® (Amersham Biosciences) and corrected using γ-actin, except for serum adiponectin determinations, which were normalized to AL.

### IR and adiponectin immunoprecipitation

10^7^ cells were plated over 75 cm^2^ and cultured with AL or CR sera for 24 hours. Cells were homogenized in lysis buffer (50 mM sodium phosphate, pH 7.4, 10% glycerol, 1% octyl phenol ethoxylate, 10 mM sodium orthovanadate, 10 mM sodium fluoride, 10 mM sodium pyrophosphate, supplemented with a Sigma protease inhibitor cocktail). After 20 min over ice, tissues lysates were centrifuged (13,000 *g*, 20 min, 4°C), and the resulting supernatants were collected. Solubilized proteins (1 mg/mL) were incubated overnight with 4 µg⋅mL^−1^ anti-IR beta subunit antibody at 4°C. Protein A-agarose (Sigma) beads (50%) were added (80 µL⋅mL^−1^), and the incubation was continued at 4°C for 2 hours. The beads were centrifuged (13,000 *g*, 1 min, 4°C), washed five times in lysis buffer and suspended in Laemmli sample buffer containing 5% 2-mercaptoethanol. Immunoprecipitation specificity was verified through SDS-PAGE separation followed by silver staining.

Adiponectin immunoprecipitation from the serum followed the same steps described above, except the serum was not diluted. The polyclonal adiponectin antibody was used at 50 µg⋅mL^−1^. After a 12 hour incubation period, protein A-agarose beads (50%, Sigma) were added (200 µL⋅mL^−1^), and the incubation was continued at 4°C for 2 hours. The beads were centrifuged (13,000 *g*, 1 min, 4°C) and the serum was analyzed by Western Blot to confirm efficiency.

### Data analysis and statistics

Data shown are representative blots or averages ± SEM of at least three identical repetitions. Data were analyzed using GraphPad Prism and compared using t-tests (for data pairs) or two-tailed ANOVA followed by Tukey tests (multiple comparisons).
